# Role of Extracellular Vesicles (EVs) in Cell Stress Response and Resistance to Cancer Therapy

**DOI:** 10.3390/cancers11020136

**Published:** 2019-01-24

**Authors:** Clodagh P. O’Neill, Katie E. Gilligan, Róisín M. Dwyer

**Affiliations:** Discipline of Surgery, Lambe Institute for Translational Research, National University of Ireland Galway (NUIG), Galway H91 YR71, Ireland; c.oneill15@nuigalway.ie (C.P.O.); k.gilligan3@nuigalway.ie (K.E.G.)

**Keywords:** extracellular vesicles, exosomes, cell stress, cancer, drug resistance, hypoxia, heat stress, biomarker

## Abstract

Extracellular vesicles (EVs) are nanosized particles released by all cells that have been heralded as novel regulators of cell-to-cell communication. It is becoming increasingly clear that in response to a variety of stress conditions, cells employ EV-mediated intercellular communication to transmit a pro-survival message in the tumor microenvironment and beyond, supporting evasion of cell death and transmitting resistance to therapy. Understanding changes in EV cargo and secretion pattern during cell stress may uncover novel, targetable mechanisms underlying disease progression, metastasis and resistance to therapy. Further, the profile of EVs released into the circulation may provide a circulating biomarker predictive of response to therapy and indicative of microenvironmental conditions linked to disease progression, such as hypoxia. Continued progress in this exciting and rapidly expanding field of research will be dependent upon widespread adoption of transparent reporting standards and implementation of guidelines to establish a consensus on methods of EV isolation, characterisation and nomenclature employed.

## 1. Introduction

As highlighted throughout this special issue, cancer cells develop mechanisms of response to conditions of stress to evade death and support tumour progression. These mechanisms support escape from apoptosis, endoplasmic reticulum (ER) stress, autophagy, hypoxia, anti-cancer therapies, heat stress and chemical stress. Understanding the mechanisms of escape from cell stress presents novel targets for therapeutic intervention and may unveil circulating biomarkers reflective of cell stress conditions and capable of predicting response to therapy. In recent years evidence has accumulated highlighting a role for extracellular vesicles (EVs) as novel mediators of these stress pathways in cancer. 

EVs are nanosized particles released by all cells and first described as far back as 1946 [1], although it is only relatively recently that their importance in normal physiology and disease has become apparent. EVs were originally considered vehicles for waste removal by cells, but are now known to contain a range of functional nucleic acids, lipids and proteins that play a key role in cell-cell communication [2]. “EV” is now the recommended universal term used for nanosized vesicles released by cells including exosomes, microvesicles (or ectosomes) and apoptotic bodies [3]. Exosomes, the smallest of the vesicles, range from 30–120 nm and are formed through the endosomal pathway and predominantly released from cells through fusion with the plasma membrane. Microvesicles/ectosomes are slightly larger, ranging from 50 nm–1 µm and are formed through direct budding from the plasma membrane. Finally, during apoptosis a large range of vesicles are released ranging from 50 nm–5 µm. No purification method specific for one EV subgroup exists, with all current approaches resulting in a heterogenous population of EVs due to overlap in size of the vesicles, along with the fact that there are no specific markers to identify or select the particular vesicle of interest [1,4]. Based on this, throughout this article vesicles reported within this broad size range in all studies will be referred to simply as EVs.

EVs contain a range of factors including proteins, lipids, RNA (mRNA, microRNA, long non-coding (lnc) RNA) and genomic DNA, and act as a reservoir protecting the material from degradation until transfer to recipient cells. EV content is thought to reflect that of the cell from which it has been released. In recent years, EVs have been implicated as a driving force in intercellular communication in the primary tumor microenvironment, mediating crosstalk that supports cancer development, adaptation to conditions of hypoxia and nutrient deprivation, escape from apoptosis and anti-cancer therapies, immune evasion and disease progression [1,5]. For example, O’Brien et al [6], demonstrated that EVs released by triple negative breast cancer (TNBC) cells were capable of increasing migration, proliferation and invasion capacity of 3 separate recipient breast cancer cell lines. EVs derived from the sera of patients with TNBC were also capable of increasing invasion of recipient cells when compared to those from healthy controls [6]. Another study highlighted the role of EVs in ovarian cancer metastasis. Highly metastatic ovarian cancer cells were found to release EVs that could induce metastatic behaviour in recipient cancer cells, and apoptosis in mesothelial cells [7]. Matrix Metalloproteinase-1 (MMP1) expression in ovarian cancer is linked with poor prognosis and was found to be packaged into EVs and transferred to the mesothelial cells [7]. Studies such as these support the hypothesis that changes in cell phenotype during conditions of stress and development of resistance to therapy may result in a change in EV cargo and pattern of release. Further, EV release into the circulation raises potential for use as circulating biomarkers reflecting changes in microenvironmental conditions and predicting response to therapy [5]. 

## 2. Response to Anti-Cancer Therapy and Transfer of Drug Resistance 

**Figure 1:**
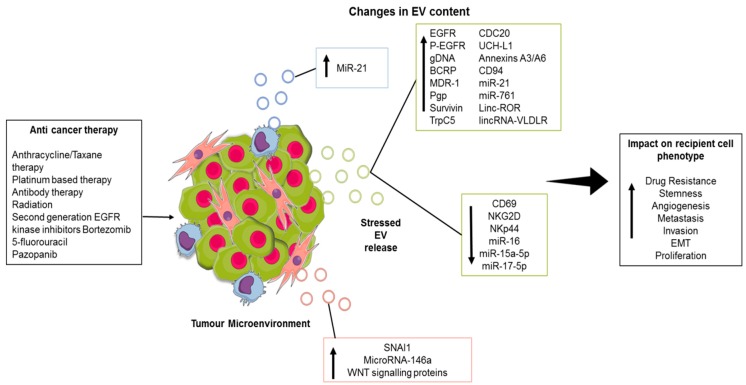
Impact of anticancer therapies on extracellular vesicle EV content and release from the tumour microenvironment. Anti-cancer therapy stress causes EV release from tumour cells and associated stromal cells including cancer-associated fibroblasts (CAFs) (pink) and tumour-associated macrophages (TAMs) (Blue). The resultant EVs have altered content including various bioactives that can have a phenotypic impact in recipient cells. This figure was created using MindtheGraph. Abbreviations: epidermal growth factor receptor (EGFR), phosphorylated-EGFR (P-EGFR), genomic DNA (gDNA), breast cancer resistance protein (BCRP), multi-drug resistance protein 1 (MDR-1), P-glycoprotein (Pgp), short transient receptor potential channel 5 (TrpC5), cell-division cycle protein 20 (CDC20), long intergenic non-protein coding RNA, regulator of reprogramming (Linc-ROR) LincRNA-very low density lipoprotein receptor (lincRNA-VLDLR), cluster of differentiation 69 (CD69), natural killer group 2 membrane D (NKG2D), activating NK receptor NKp44 (NKp44), snail family transcriptional repressor 1 (SNAI1).

A number of studies have demonstrated a change in the content of EVs secreted by tumour cells in response to chemotherapy and radiation-induced cell stress, resulting in transfer of a resistant phenotype and immune evasion, and raising potential for this altered profile as a biomarker of response or resistance to therapy (Figure 1). Potential novel therapeutic targets have also been highlighted through study of altered EV content in this setting. 

Evidence of EV-mediated transfer of drug resistance in response to cell stress in vitro continues to accumulate in a range of cancers including pancreatic [8], prostate [9], breast [10,11,12], ovarian [13], osteosarcoma [14], non-small cell lung cancer [15] melanoma [16] and acute myeloid leukaemia [17].

Recently, BRAF inhibitor resistant melanoma cells were shown to activate a novel truncated form of anaplastic lymphoma kinase (ALK), which was secreted in EVs that were then capable of transferring drug resistance through activation of the mitogen-activated protein kinase (MAPK) signalling pathway in recipient cells [16]. The ALK protein cargo in EVs following irradiation was also implicated in driving tumour growth and compromising therapeutic efficacy of ALK inhibitors in non-small-cell lung carcinoma (NSCLC) [18]. EVs isolated from ALK-containing H3122 cells following irradiation contained much higher levels of phosphorylated ALK (p-ALK) than control EVs, and activated AKT, STAT3, and the ERK pathway in recipient cells [18]. 

Following irradiation, GBM cells were also shown to promote proliferation and therapy resistance of surviving tumour cells by secreting apoptotic extracellular vesicles (apoEVs) enriched with various components of spliceosomes [19]. ApoEVs altered RNA splicing in recipient cells, partly through transfer of splicing factor RBM11, resulting in more oncogenic isoforms of MDM4 and Cyclin D1 thereby promoting therapy resistance and an aggressive migratory phenotype [19]. The protein cargo of EVs released from head and neck squamous cell carcinoma in vitro was also found to reflect changes in cellular processes induced by radiation e.g., transient suppression of transcription/translation and stress-induced signalling [20]. Indeed, investigation of the p53-mediated response following lung cancer cell irradiation revealed the protein TSAP6 enhanced EV production in cells, highlighting a novel function of the p53 protein in regulation of EV secretion [21].

Exposure to heat shock and chemotherapy drugs caused a significant increase in EV release by hepatocellular carcinoma (HCC) cells in vitro, with the secreted vesicles enriched with biologically active heat shock proteins (HSP60, HSP70, HSP90) and capable of stimulating an increase in cytotoxic activity of NK cells [22]. Also in HCC, changes in EV encapsulated long non-coding RNAs have been demonstrated in response to anticancer drugs, unveiling potential novel mechanisms involved in response to therapy and pathways towards acquired chemoresistance [23,24]. 

Breast cancer cells were shown to release EVs enriched with the cell survival protein Survivin following exposure to microtubule inhibiting drugs such as Paclitaxel, resulting in increased drug resistance and survival of recipient cells [25]. EVs from docetaxel-resistant prostate cancer cells were capable of transferring resistance to recipient cells, potentially due to EV Multidrug resistance-1 (MDR-1)/P-glycoprotein (P-gp) transfer preventing accumulation of drugs within cells through active drug efflux [9]. EVs from prostate cancer patients’ sera were also demonstrated to induce increased cell proliferation and invasion, compared to EVs from age-matched controls [9]. 

Keklikoglou et al. [26] demonstrated an increase in the number of EVs released from cancer cells following exposure to two common breast cancer therapies, PTX and doxorubicin (DOX). Further, the EVs released from treated cells had an enhanced pro-metastatic capacity. Using immunocompetent animal models, the study demonstrated that injection of EVs derived from chemotherapy treated cells resulted in an increase in metastatic lung nodules. This pro-metastatic capacity was also confirmed in a zebrafish embryo model. Proteomic analysis revealed enrichment of the cell signalling protein annexin A6 (ANXA6) in the EVs, which was demonstrated to play a key role in the effects seen. ANXA6 was also detected in circulating EVs of breast cancer patients undergoing neoadjuvant chemotherapy, raising potential as a circulating biomarker of disease progression or response to therapy [26]. 

EVs released by cells within the tumour microenvironment in response to anti-cancer therapies have also been shown to mediate resistance. Following treatment with 5-fluorouracil, mesenchymal stem cell (MSC)-derived EVs had a protective effect on gastric cancer cells, reducing chemotherapy induced apoptosis through activation of the calcium/calmodulin-dependent protein kinases (CaM-Ks) and Raf/MEK/ERK kinase signalling cascade [27]. EVs derived from bone marrow mesenchymal stromal cells (BM-MSC) were also demonstrated to increase chronic lymphocytic leukemia B cell chemoresistance to several drugs, enhance migration and decrease apoptosis [28]. Gemcitabine treatment of carcinoma associated fibroblasts (CAFs) stimulated release of EVs capable of promoting proliferation and chemoresistance of pancreatic ductal adenocarcinoma cells [29]. The microRNA content of EVs released by gastric tumour-associated macrophages (TAMs) was also altered following cisplatin treatment [30], with increased EV-miR21 transferred to recipient gastric cancer cells, suppressing apoptosis and activating the PI3K/AKT signalling pathway through down regulation of PTEN [30]. These studies highlight the possibility that novel treatment strategies targeting EV-mediated intercellular communication prior to chemotherapy may be effective in reducing this pro-tumorigenic signalling.

In addition to mediating transfer of microRNA/mRNA/lncRNA and proteins to confer a drug resistant phenotype upon recipient cells, EVs have also been shown to act as drug decoys, thus shielding target tumour cells. B-cell lymphoma-released EVs were demonstrated to have elevated CD20 embedded in the membrane which acted as a decoy target, intercepting and binding to rituximab [31]. Transient receptor potential channel 5 (TrpC5) has also been shown to accumulate in EVs released by drug resistant breast cancer cells, resulting in sequestration of chemotherapeutic drugs [32]. Transfer of EV-TrpC5 to recipient cells was demonstrated to result in transfer of resistance. Further, the level of circulating EV-TrpC5 had a significant positive association with TRPC5 protein level in tissues of patients with advanced breast cancer prior to chemotherapy, suggesting a role for circulating EVs in evaluation of chemoresistance [33].

Further studies using patient samples have demonstrated that along with influencing a chemoresistant phenotype, EVs have potential as circulating biomarkers of resistance or response to chemotherapy, which may play an important role in the clinical decision making process. Breast cancer resistance protein (BRCP) was found to be upregulated at the mRNA and protein level in tumour derived circulating EVs from patients with a poor response to chemotherapy [34]. MUC1 was also co-expressed with BCRP, raising potential as a predictive biomarker of response to chemotherapy [34]. A strong negative correlation between the level of ubiquitin carboxyl terminal hydrolase-L1 (UCH-L1) in circulating EVs and the clinical outcome of chemotherapy has also been reported, highlighting potential for monitoring chemotherapeutic efficacy [10].

Yin et al. [35] reported increased annexin A3 in sera of platinum resistant ovarian cancer patients, raising potential as a biomarker of resistance to platinum-based therapies. In Vitro, ovarian cancer cells expressing higher levels of annexin A3 released increased numbers of EVs, with the protein detected in EVs from cisplatin-resistant cells [35]. In patients with multiple myeloma, downregulation of a panel of microRNAs (miR-16, miR-15a, miR20a and miR-17) was associated with bortezomib resistance [36]. Also focused on microRNA content, analysis of EVs released by pazopanib treated/untreated synovial sarcoma cells in vitro highlighted EV encapsulated miR-761 as a potential biomarker of pazopanib resistance [37]. Using cell lines from a range of cancer types, Montermini et al [38] demonstrated that treatment with second generation EGFR kinase inhibitors stimulated release of EVs containing EGFR, phosphorylated EGFR, and genomic DNA. The data presented suggests that the EV profile could be used as a surrogate indicator of drug-related stress response [38]. 

EVs have also been implicated in the mechanism of action of drugs in development. Shiau et al. [39], showed that the phytoagent deoxyelephantopin (DET) and its chemical derivative DETD-35 suppressed triple negative breast cancer (TNBC) in vitro and in vivo. DET and DETD-35 treatment induced mitochondrial dysfunction and downstream oxidative stress. Interestingly EVs released from the TNBC cells after DET/DETD-35 treatment had anti-tumour phenotypes i.e., reduced expression of proteins involved in cell migration, adhesion and angiogenesis. This study supports the suitability of DET/DETD-35 as a potential anti-cancer drug, to treat TNBC partly through emission of anti-cancer EVs [39]. 

Suberoylanilide hydroxamic acid (SAHA), is a histone deacetylase inhibitor that has anti-cancer drug potential. Campanella et al. [40], studied the mechanism of action of this chemical in lung carcinoma cells in vitro. Similar to DET/DETD-35 [39], treatment of cancer cells with SAHA, caused mitochondrial damage and oxidative stress. This chemical stimulated release of EVs containing high expression of a nitrated version of heat shock protein 60 (HSP60). It was postulated that EV-nitrated Hsp60 could interact with the immune system, but its anti-cancer properties are yet to be fully elucidated [40].

## 3. Hypoxia

Low oxygen tension or hypoxia is often present in tumour tissue, and this is associated with more aggressive cancers and poor patient prognosis. The tumour microenvironment is heterogeneous, with varying levels of hypoxic conditions throughout. Cancer cells have acquired many mechanisms to cope with this environmental stress, with the most well-known mediators being the hypoxia inducible factors (HIF) including HIFα and HIFβ. These proteins can activate downstream signalling pathways relating to tumour growth, metastasis and angiogenesis. The release of EVs in response to hypoxia has been reported in various cancers including breast, prostate and multiple myeloma [41,42,43,44,45,46]. EV release provides a novel hypoxia response mechanism facilitating transformation of the microenvironment into a cancer promoting utopia, with many studies showing EV involvement in transfer or regulation of HIF proteins (Table 1) [43,44,45,46,47]. 

Hypoxia resistant multiple myeloma (HR-MM) cells, generated through continuous exposure to hypoxic conditions in vitro, were shown to have a two fold increase in EV release compared to the parent MM cells [43]. Promotion of angiogenesis by EV-miRNA from HR-MM cells was also demonstrated, mediated through miR-135b suppression of the factor-inhibiting hypoxia-inducible factor 1 (FIH-1) [43]. EVs released from leukemia cells cultured in hypoxic conditions were also shown to stimulate a significant increase in human umbilical vein endothelial cells (HUVEC) tubule formation in vitro compared to EVs from control cells [46]. Kucharzewska et al. [48] showed that under hypoxia conditions GBM cell secreted EVs that induced growth factor and cytokine release by recipient endothelial cells, in turn activating PI3K/AKT signalling and migration of pericytes. A range of breast cancer cell lines exposed to varying conditions of hypoxia in vitro have also been shown to release increased numbers of EVs with increasing hypoxia [44,45].

EVs released from hypoxic oral squamous cell carcinoma (OSCC) cells initiated cell migration and metastasis in recipient cells through HIF-1α and HIF-2α expression [49]. miRNA analysis of the EVs highlighted miR-21 as being significantly elevated compared to EVs released in normoxic conditions. Functional analysis revealed that miR-21 promoted invasion and migration of recipient cells in a HIF-1α and HIF-2α dependent manner [49]. Further, EV release by breast cancer cells was reported to be HIF dependent and mediated by expression and co-localisation of shedding EVs with RAB22A, a small GTPase. Incubation of breast cancer cells with these EVs resulted in increased focal adhesion formation and ECM invasion in vitro and promoted extravasation in vivo. In hypoxic conditions prostate cancer cells were also shown to release EVs enriched with signalling molecules that were capable of promoting invasiveness of normal prostate cancer cells [50]. Long non-coding RNAs (lncRNAs) have in recent years been shown to play a role in regulation of gene expression. Long intergenic noncoding RNA, regulator of reprogramming (linc-ROR) was shown to be highly enriched in EV fractions from HCC cells after hypoxia exposure. Hypoxia-induced EVs promoted cell survival at least in part through Linc-ROR mediated regulation of the HIFα pathway in HCC cells [47].

Given the mounting evidence for the role of EVs in cellular response to hypoxia, a number of groups have investigated the potential of circulating EVs as biomarkers of hypoxia. For example, miR-210 expression has been reported as upregulated in EVs released from multiple myeloma, leukaemia, and breast cancer cells in hypoxic conditions [43,45,46]. Li et al., [49] investigated the use of EV-miR-21 as a biomarker in OSCC. EVs were isolated from the plasma of OSCC patients (*n* = 108) and healthy controls (*n* = 108). Circulating EV-miR-21 was shown to be significantly higher in OSCC patients than healthy controls, and the levels were found to be associated with clinical characteristics such as tumor Stage and lymph node metastasis. Through HIF-1α and HIF-2α staining, EV-miR-21 was also linked to tumor hypoxia [49]. EVs released from glioblastoma (GBM) cells during hypoxia have been shown to be enriched in hypoxia regulated proteins and mRNA including caveolin 1 (CAV1), interleukin-8 (IL8), platelet-derived growth factor (PDGF) and MMPs [48]. The study raised the potential for these EV molecules as a signature of oxygenation status and aggressiveness of GBM tumours. Lipid accumulation in EVs derived from prostate cancer cells exposed to hypoxia has also been postulated to have biomarker potential to assess tumor oxygenation status and aggressiveness [41]. EVs from the hypoxic prostate cancer cells were found to have increased accumulation of triglycerides. After reoxygenation these lipids supported rapid prostate cell growth. Blockade of lipid formation by various drugs including the COX2 inhibitor celecoxib, reduced tumour growth and invasiveness after reoxygenation, suggesting a potential therapeutic target for prostate cancer treatment [41].

As previously highlighted, EVs play a key role in mediation of tumour-stroma interactions. In the hypoxic tumour environment, tumour-associated macrophages (TAMs) lose their anti-tumour phenotype and are linked with much poorer outcomes in cancer patients. Hsu et al. [52], showed a role for EVs in amplifying the macrophage oncogenic effects in lung cancer under hypoxic stress. Incubation of M2 macrophages with EVs released by hypoxic lung cancer cells reprogrammed the macrophages towards a pro-tumorigenic, immunosuppressive phenotype through EV-miR103a signalling [52]. Tumour-derived EVs have also been shown to interact with NK cells under hypoxic conditions. Through both in vitro and in vivo experiments EVs from hypoxic tumour cells were shown to impair NK cell cytotoxicity through the transfer of proteins including TGF-β1, and miRNA including miR-210 and miR-23a [51].

## 4. Nutrient Deprivation 

Due to the rapid increase in cell growth in the tumour microenvironment, nutrient deprivation is a prevalent stress. MSCs are mass producers of EVs and have been shown to survive well under nutrient starvation stress [53,54]. Vallabhaneni et al., [54] investigated the cargo of EVs from serum-deprived MSCs (SD-MSCs) associated with the tumour environment. Proteomic, nucleic acid, and lipid analysis of the EV cargo was performed. EV lipid analysis confirmed the presence of bioactive lipids with pro-tumourgenic characteristics. MiRNA analysis identified miR21 and miR34a as key oncomiRs, with roles in tumour progression and proliferation confirmed in vitro and in vivo [54]. 

Following on from this study, in 2016 the same group studied the role of these nutrient deprived MSC-EVs in osteosarcoma (OC) [53]. OC cells incubated with EVs from SD-MSCS showed resistance to apoptosis and increased wound healing in vitro. The recipient cells were found to express miRNAs that could potentially target metabolism and metastasis associated genes. Alteration in expression of target genes including matrix metalloproteinase (MMP1) and focal adhesion kinase (PTK2) was validated by qPCR [53]. 

## 5. ER Stress and Apoptosis

The endoplasmic reticulum (ER) is essential in maintaining cell homeostasis, however under stressful conditions cells induce an unfolded protein response (UPR). ER Stress has been linked to multivesicular body (MVB) formation, and increased EV release. This increased EV release was only found in cells containing ER stress transducers inositol required enzyme 1 (IRE1) and PKR-like ER kinase (PERK) [55]. Interestingly ER stress has also been found to be induced by EVs. Tumour-derived EVs containing miR-3091-3p internalised by hepatocytes suppressed autophagy-related protein 9b (Atg9b) expression. This led to ER stress-induced cell death by accumulation of ubiquitinated proteins [56]. Javeed et al., [57] found that pancreatic cancer shed adrenomedullin+/ CA1909+ EVs. The EVs then induced ER stress along with failure of the UPR causing paraneoplastic b-cell dysfunction resulting in inhibited insulin secretion. 

If cell stress cannot be relieved by UPR and DNA damage response to maintain equilibrium, cell death is initiated [58]. Pavlyukov et al., [19] showed in GBM cells the paradoxical role of apoptotic cell derived EVs (ApoEVs) in promoting tumour growth and drug resistance in neighbouring cells, demonstrated in vitro and in vivo. Analysis of the ApoEVs uncovered that proteasomal and spliceosomal proteins were significantly enriched compared to control GBM EVs. These spliceosome related proteins were taken up by target cells and had an effect on mRNA splicing. Through this process a more aggressive and therapy-resistant phenotype was promoted in surviving cells [19].

## 6. Autophagy 

Autophagy allows the breakdown of unnecessary or damaged proteins and organelles, in order to maintain cell homeostasis. Under stress conditions autophagy is increased, and dysregulation of this highly conserved mechanism is linked to diseases including cancer [59,60]. Recent reports have demonstrated an important connection between EVs and autophagy during stress in the context of cancer [60].

Bhattacharya et al., [61] reported the interlinking role of autophagy and EV biogenesis in pancreatic cancer. G alpha interacting protein (GAIP) C-terminus (GIPC) was shown to be a master regulator of autophagy, with down regulation resulting in decreased glucose uptake in pancreatic cancer cells leading to metabolic stress. This stress in turn led to autophagy induction and an increase in the number of EVs released [61]. The chemical pesticide rotenone was also shown to induce chemical stress in cancer stem cells from both prostate and breast cancer cell line, resulting in extensive mitochondrial damage and induction of autophagy [62]. This in turn led to increased EV formation and release.

Interestingly, Dutta et al., [63] have shown that EVs released from breast cancer cells induced an increase in reactive oxygen species (ROS) in recipient human mammary epithelial cells (HMECs), leading to increased autophagy. These EV-treated HMECs also released growth factors promoting further tumorigenesis [63]. 

## 7. Heat Stress and Oxidative Stress 

Heat stress has been found to influence EV release. Bewicke-Copley et al, [64] found that EVs released by heat shocked cells induced a bystander damage in unstressed populations. DNA damage and apoptosis was increased with cell viability reduced. While this could be a positive outcome in the cancer setting it was discovered that cells treated with EVs from heat shocked cells were more likely to survive a subsequent heat shock. This could mean that the EVs allowed the cells to develop an adaptive response and become resistant to hyperthermia therapies. Oxidative and heat stress have also been implicated in blood malignancies. The reason for relapse and poor prognosis in blood cancer is thought to be associated with inadequate NK-Cell function. Two lymphoma cell lines were found to release EVs bearing the NKG2D ligands MICA/B and ULBP1 and 2. These EVs were found to impair NK-Cell function by NKG2D receptor mediated cytotoxicity. When heat stress was applied EV secretion was enhanced, generating more soluble NKG2D ligands, aggravating impairment of the cytotoxic response. This is another consideration that should be taken into account when designing hyper-thermal anti-cancer therapies [65]. This study shows the role of oxidative and heat stressed EVs in this process [65], and supports the previously mentioned role of EVs from hypoxia treated tumour cells in NK cell cytotoxicity [51].

In contrast to these studies, heat stress was found to have a positive effect on the immunogenicity of EVs released by heat-treated ascites. The EVs were able to induce a tumour-specific cytotoxic T lymphocyte response and promote dendritic cell maturation [66]. Another study found that EVs derived from dendritic cells or tumour cells could induce specific anti-tumour immunity. Under hyperthermic conditions it was found that EVs derived from heat-stress treated tumour cells (HS-TEX) could activate dendritic and T cells, when compared to untreated cells. Intra-tumoural injection of the HS-TEX induced an anti-tumour immune response inhibiting tumour growth and increasing animal survival time [67]. Guo et al, [68] found similar results with HS-TEX in that they could induce an anti-tumour response. HS-TEX contained the ability to convert immunosuppressive regulatory T cells into T helper type 17 cells via interleukin-6 (IL-6). IL-6 promotes IL-17 expression which in turn causes rejection of established tumours. The ability of heat stress to aid in the release of EVs carrying drugs was investigated by Yang et al, [69]. The study examined whether heat stress facilitated in MCF-7 doxorubicin (DOX) cells releasing more EVs containing the drug. It was found that more EVs were released from the heat stress cells and that they also contained higher levels of DOX than non-heat stress cells of the same number. The EVs also inhibited MCF-7 proliferation and induced cell apoptosis in vitro along with inhibiting tumour growth in vivo. 

## 8. Conclusions

Due to the recent interest in EVs in the cancer setting, the number of publications in this field has increased dramatically over the past decade. However, issues with interpretation of studies and reproducibility have arisen due to the different methods of isolation, characterisation and nomenclature employed. Table 1 highlights some of the EV isolation and characterisation methods routinely employed. Although novel approaches to improve EV samples purity and yield are constantly in development, ultracentrifugation remains the most frequently employed method, isolating EVs using speeds >100,000× *g* for >1 h [70,71]. This is often preceded by differential centrifugation at lower speeds to remove cell debris and proteins aggregates, and microfiltration. Many investigators couple a density based separation technique with a size exclusion isolation technique to improve the purity of samples. In recent years commercial EV isolation kits have also come on the market, however there are concerns that the isolated EVs may be accompanied by contaminating factors [71]. Refined isolation techniques are emerging e.g., tangential flow filtration [72], with the aim of supporting isolation of high yields of good manufacturing practice (GMP)-grade EVs for use in the clinical setting [73]. In the past, based on the information available at the time, researchers were ascribing functions to a specific EV type despite the fact that they were working with a heterogenous population, potentially containing vesicles of mixed origin (exosomes/microvesicles/apoptotic bodies) along with co-purified bound material in some cases [74]. This lead to the publication of the Minimal Information for Studies of Extracellular Vesicles (MISEV) 2014 [75]. This described a “set of biochemical, biophysical and functional standards that should be used to attribute any specific biological cargo or functions to EVs”. As the field continued to rapidly evolve and expand, in 2017 EV-TRACK was published recommending that authors report specific experimental parameters for EV isolation and characterisation, to increase transparency and support interpretation of data presented and reproducibility of findings [76]. This included the “EV-METRIC”, a checklist of information that should ideally be reported for EV experiments including: methods of isolation such as density gradient, EV density or Ultracentrifugation; protein analysis targeting ≥3 EV-enriched proteins, along with a negative control protein and the lysis buffer composition; and both quantitative and qualitative particle analysis should be performed including wide field and close up electron microscopy imaging [76]. 

Following consultation with investigators in the field of EV research, in 2018 updated MISEV guidelines were published [3]. This includes the reporting of EV isolation procedures in detail, with refinement of culture and harvesting conditions e.g., dead cell count in cultures should now be provided by investigators. For EVs from biological fluids and tissues, guidance on methods of collection and storage has been provided, along with the noting of clinicopathological details that could have an impact on the sample. If a study claims that an effect seen is caused by EVs, it should demonstrate that the function is EV specific, and not a result of cell-cell contact or other soluble mediators. Overall MISEV2018 is aiming to standardise the quality of the research being conducted by allowing more reliable and reproducible results [3]. Continued progress in this exciting and rapidly expanding field of research, using EVs to unlock novel mechanisms of cancer progression and resistance to therapy will be dependent on widespread acceptance and implementation of these guidelines.

## Figures and Tables

**Table 1:** Impact of hypoxia on EV release and content. NTA: Nano Particle Tracking Analysis; TEM: Transmission Electron Microscopy; WB: Western Blot; HIF: Hypoxia Inducible Factor; TRPS: Tunable Resistive Pulse Sensing; TD-EV: Tumor Derived Extracellular Vesicles; TGF: Transforming Growth Factor.

Cancer Type	EV Isolation Technique	EV Characterization	EV Content Change under Stress	Study Outcome	Reference
Prostate	Ultracentrifugation Or commercially available kit	NTA	Prostate cancer (PCA) cells released EVs enriched in triglycerides due to activation of lipogenesis-related enzymes and signalling molecules	Significance of lipid accumulation in hypoxic PCA cells and EVs; therapeutic relevance in PCA.	[41]
Prostate	Ultracentrifugation Or commercially available kit	NTA TEM WB	Higher number of proteins in EV (Hypoxic) compared to EV (Normoxic), primarily associated with the remodelling of epithelial adherens junction pathway	EV (Hypoxic) are loaded with proteins that could enhance invasiveness, stemness and induce microenvironment changes, thereby promoting PCA aggressiveness	[50]
Multiple Myeloma (MM)	Commercially available kit	TEM NTA	miR-135b was significantly upregulated in EVs from Hypoxia resistant (HR) MM.	HR-MM cells may mimic in vivo bone marrow microenvironment. EV-miR-135b potential target for controlling angiogenesis	[43]
Leukaemia	Commercially available kit	TEM NTA WB	Range of miRNAs including miR210 significantly increased in EVs secreted from hypoxic K562 cell line	EV-miRNA derived from cancer cells under hypoxic conditions may influence angiogenic activity in endothelial cells	[46]
Breast	Differential centrifugation	NTA	Increased number of EV particles released in HIF-dependent manner. RAB22A protein co-localizes with budding EVs.	EVs can induce complex cytoskeletal alterations in a RAB22A-dependent manner promoting Breast cancer metastasis	[44]
Breast	Ultracentrifugation Or commercially available kit	TEM NTA WB	Increase in EV-miR-210 released from breast cancer cells	Hypoxia promoted the release of cancer EVs containing elevated miR-210	[45]
Hepatocellular cancer (HCC)	Ultracentrifugation	TEM NTA Protein yield	Long intergenic noncoding RNA (linc-RoR) was incorporated into EVs.	Linc-RoR enriched EVs released by tumour cells during hypoxia modulated recipient cell signaling and survival	[47]
Glioblastoma multiforme (GBM)	Ultracentrifugation	NTA WB	The proteome and mRNA profiles of EVs reflected the oxygenation status of GBM cells and patient tumors.	>EV pathway constitutes a potentially targetable driver of hypoxia-dependent intercellular signaling. EVs signature holds biomarker potential.	[48]
Multiple Tumor Types	Ultracentrifugation	TRPS TEM	Hypoxic TD-EVs transferred TGF-β1. Increased EV-miR-210 and EV-miR-23a.	Novel mechanism of immune suppression mediated by hypoxic TD-EVs	[51]
Lung	Commercially available kit	WB	Increased levels of EV-miR-103a.	EV-miR-103a enhanced tumor progression and angiogenesis. Increased miR-103a levels seen in lung cancer patients.	[52]
Oral Squamous cell carcinoma (OSCC)	Commercially available kit	SEM WB	108 OSCC EV-miRNAs were differentially expressed, miR-21 significantly upregulated.	Hypoxic microenvironment may stimulate tumor cells to generate miR-21-rich EVs that are delivered to normoxic cells to promote prometastatic behaviors.	[49]
